# Essential Pathology of Cancer

**Published:** 1844-08-10

**Authors:** 


### ESSENTIAL PATHOLOGY OF CANCER.

From the researches of Müller it would appear that in the formation of carcinomatous structures "an arrest of development took place, and the primitive cells, instead of progressing in the construction of the more complex tissues of the body, took upon themselves the function of reproducing other cells, thus constituting, by their aggregate, a mass analogous to what we find forming the entire fabric of some of the lowest beings amongst plants and animals."—*Dr. Power, in Dublin Medical Press.*

